# Preparation of ZnCl_2_-Activated Magnetic Biochar and Its Performance in Removing Hexavalent Chromium from Water

**DOI:** 10.3390/nano15201586

**Published:** 2025-10-17

**Authors:** Pingqiang Gao, Zhe Tan, Yonghao Yan, Min Yang, Shuai Han, Chen Yang, Shuai Li, Yan Zhang

**Affiliations:** 1 School of Chemistry and Chemical Engineering, Yulin University, No. 51 Chongwen Road, Yulin 719000, China; gaopingqiang@yulinu.edu.cn (P.G.); tanzhe@stu.yulinu.edu.cn (Z.T.); yanyonghao@stu.yulinu.edu.cn (Y.Y.); yangmin@stu.yulinu.edu.cn (M.Y.); hanshuai@stu.yulinu.edu.cn (S.H.); yangchen@stu.yulinu.edu.cn (C.Y.); lishuai@stu.yulinu.edu.cn (S.L.); 2 Yulin Engineering Research Center of Coal Chemical Wastewater, Yulin University, No. 51 Chongwen Road, Yulin 719000, China

**Keywords:** jujube branches’ magnetic biochar, ZnCl_2_ activation, Cr(VI), removal mechanism

## Abstract

Magnetic biochar (Zn/Fe-BC) was prepared from jujube branches via an impregnation pyrolysis–coprecipitation technique to eliminate Cr(VI) from water. ZnFe_2_O_4_ was introduced through ZnCl_2_-based impregnation and pyrolysis, which can regulate the microstructure of hydrocarbon frameworks. Furthermore, FeSO_4_·7H_2_O was used as the precursor for co-precipitation to embed Fe_3_O_4_ into the material, improving its reducibility and magnetism. The results demonstrated that Zn/Fe-BC exhibited excellent Cr(VI) removal efficiency. Under optimal conditions (an initial Cr(VI) concentration of 50 mg/L, pH 2, and an adsorbent dosage of 2 g/L), the maximum adsorption capacity of Zn/Fe-BC reached 27.85 mg/g, which was significantly higher than that of unmodified biochar (23.20 mg/g). Following five cycles of adsorption and desorption, the desorption efficiency was still higher than 60.35%. The following were the inhibitory effects of coexisting anions on the elimination of Cr(VI): CO_3_^2−^ > PO_4_^3−^ > SO_4_^2−^ > NO_3_^−^. According to kinetic and isothermal adsorption experiments, the adsorption process adhered to the Freundlich isotherm and followed a pseudo-second-order kinetic model, indicating a multilayer adsorption process. Cr(VI) removal by Zn/Fe-BC was driven by physical adsorption and chemical reduction, involving a synergistic combination of electrostatic attraction, reduction, complexation, precipitation, and pore filling. These findings demonstrate the potential of the Zn/Fe-BC magnetic biochar as an effective adsorbent for Cr(VI) remediation in water treatment applications.

## 1. Introduction

With the acceleration of industrialization and urbanization, water resource scarcity and water pollution have gradually become key concerns of the public [1]. Chromium (Cr) is an important metal element widely present in nature and industrial processes. Several industries, including printing and dyeing, tanning, electroplating, and medical, use it extensively [2]. In water, chromium mostly exists in the valence states Cr(III) and Cr(VI). Among them, Cr(VI) has stronger toxicity and mobility than Cr(III) [3]. Once it enters the environmental system, Cr(VI) will pose a long-term threat to the ecological environment and human health [4]. Therefore, the removal of Cr(VI) in water has become an urgent problem to be solved. The adsorption method is considered to be a feasible and economical method for removing pollutants because of its safety, simplicity, high efficiency, and strong adaptability [5]. The commonly used adsorbents are activated carbon, carbon nanotubes, zeolite, and kaolin. However, the adsorption materials mentioned above have problems such as high price, low adsorption efficiency, and difficult recovery in the process of practical application [6,7,8].

Carbon-rich biochar is inexpensive and readily accessible. It is made by pyrolyzing biomass in anoxic conditions, usually at temperatures between 300 and 700 °C [9]. Because of its extensive specific surface area, high aromatic content, stable porous framework, and plentiful oxygen-containing functional groups, biochar is considered a promising carbon-based adsorbent. In China, jujube branches, an agricultural byproduct, are produced in large quantities annually, but they are rarely utilized effectively. Converting these branches into biochar not only provides a sustainable strategy for solid waste reduction but also promotes resource recovery and utilization. Composed mainly of lignin, cellulose, hemicellulose, and minerals, jujube branches can produce porous carbon materials with excellent adsorption properties after pyrolysis activation. Pine needle biochar, which is rich in lignocellulose, has an adsorption capacity of 4.18 mg/g for Cr(VI), according to Zhang et al. [10]. Dahiya et al. [11] reported that dynamic functional groups, including lactones, carboxylic esters, and phenols, exert a significant impact on Cr(VI) adsorption onto biochar besides surface area and surface charge. Furthermore, Choi et al. [12] used a new corolla-like MnO_2_-decorated porous magnetic biochar to report successful adsorption of Cr(VI) and As(V).

Despite its advantages, raw biochar often exhibits limited adsorption capacity and functionality. Thus, this study aims to enhance the porosity, functional group concentration, and specific surface area of biochar by applying activation treatments [13]. In biochar alteration, common chemical activators include metal-based chemicals, HCl, KOH, and NaOH. Among these, zinc chloride (ZnCl_2_) is considered a promising activator. Due to its low melting point (263 °C), ZnCl_2_ allows cellulose to expand in its liquid state and penetrate the macromolecular structure, promoting the hydrolysis of hydroxyl groups in organic matter and the rearrangement of carbon molecules [14]. Additionally, the relatively low boiling point of ZnCl_2_ (732 °C) makes it easier for elemental Zn and ZnCl_2_ to be released as gases during pyrolysis, which encourages the formation of microporous and mesoporous structures in the biochar [15]. This modification enhances the material’s ability to adsorb contaminants like tetracycline, As(III), and Cr(VI). For instance, according to Ding et al. [16], vinegar residue modified with ZnCl_2_ has a much higher capacity for chromium adsorption (236.81 mg/g) than unmodified acidic vinegar residue (9.96 mg/g). Zheng et al. [17] used traditional Chinese medicine residue to prepare ZnCl_2_-activated biochar via a one-pot method. More active sites for Cr(VI) adsorption were made possible by the activated biochar’s 111.98-fold increase in specific surface area, which reached 145.13 m^2^/g. Huang et al. [18] developed ZnCl_2_-modified corncob biochar for benzene adsorption from air, displaying a maximum capacity for adsorption of 170.53 mg/g. These studies confirm the effectiveness of ZnCl_2_ as an effective biochar modifier.

In addition to chemical activation, biochar doping with metals such as FeCl_3_, nano-zero-valent iron, cobalt (Co), and nickel (Ni) is another efficient strategy to improve its adsorption performance. This strategy improves the surface functional group density and internal structure of biochar, increasing its adsorption capacity [19]. Fe doping is regarded as a promising approach among various metal doping techniques because of its affordability, effectiveness, and environmental friendliness. This modification endows biochar with excellent magnetic properties, high adsorption activity, and good stability, making it particularly suitable for remediating heavy metal pollution and treating organic wastewater [20]. Wang et al. [21] demonstrated through a 1,10-phenanthroline shielding experiment that Fe(II) contributes 17.6% to Cr(VI) removal. Campos et al. [22] synthesized a core–shell dual magnetic nano-adsorbent (S_BET_: 152.6 m^2^/g; Q_max_: 15.6 mg/g). Sangkarak et al. [23] synthesized a magnetic powdered activated carbon that has high magnetic characteristics (saturation magnetization of 9.66 emu/g) and an outstanding Cr(VI) adsorption capacity (Langmuir adsorption capacity of 75.76 mg/g). In summary, metal doping, especially Fe-based modification, has shown significant potential in enhancing the adsorption capabilities of biochar.

Although ZnCl_2_ activation and Fe doping have shown their respective advantages, the synergistic application of the two modification strategies to single biochar materials, especially using waste jujube branches as raw materials, still lacks systematic research. This synergistic modification is expected to integrate the dual advantages of high adsorption capacity and easy magnetic separation, and overcome the limitations of traditional adsorbents and single modified biochar. Based on this, in this study, a novel biochar, Zn/Fe-BC, was prepared via co-pyrolysis of jujube branches with ZnCl_2_, followed by coprecipitation with FeSO_4_·7H_2_O. The adsorption performance, mechanism, and practical application of Zn/Fe-BC for Cr(VI) removal were investigated through adsorption experiments and various characterization techniques, including SEM, BET, XRD, XPS, Raman spectroscopy, and FT-IR spectroscopy. The primary goals for this study are to (1) create and describe the Zn/Fe-BC composite; (2) assess how environmental factors (biochar dosage, solution pH, interference from coexisting ions, and reusability) on the Cr(VI) removal efficiency of Zn/Fe-BC; and (3) clarify the mechanism behind Cr(VI) removal by Zn/Fe-BC through kinetic, isotherm, and thermodynamic modeling, along with FT-IR and XPS analyses.

## 2. Materials and Methods

### 2.1. Materials and Reagents

The jujube branches utilized in this research were gathered as agricultural waste from Yulin City, Shaanxi Province, China. The chemical reagents employed included ZnCl_2_, FeSO_4_·7H_2_O, NaOH, HCl, H_2_SO_4_, H_3_PO_4_, KCl, NaCl, MgCl_2_, CaCl_2_, Na_2_CO_3_, Na_3_HPO_4_, NaNO_3_, Na_2_SO_4_, diphenylcarbazide, acetone, and potassium dichromate. Tianjin Shengao Chemical Reagent Co., Ltd. (Tianjin, China) provides all of the analytical-grade reagents.

### 2.2. Preparation of Zn/Fe-BC

**Figure 1:**
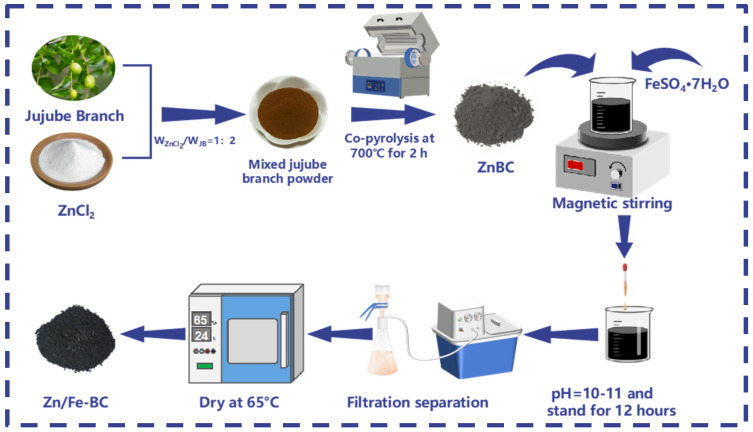
The preparation process of Zn/Fe-BC.

Figure 1 illustrates the preparation process of Zn/Fe-BC. First, jujube branches were thoroughly washed with deionized water to remove surface contamination. The cleaned samples were then dried in an oven at 65 °C for 24 h. The dried material was ground through a 150-mesh sieve and stored for further use. The jujube branch powder was subsequently mixed with ZnCl_2_ at a mass ratio (W_ZnCl2_/W_JB_) of 1:2, thoroughly stirred, and soaked completely with magnetic stirring for 4 h. After drying, the mixture was once again ground. The following parameters were used to pyrolyze the dried mix in a tube furnace: a target temperature of 700 °C, 2 h, 5 °C/min, and 50 mL/min. The resultant ZnCl_2_-modified biochar (ZnBC) was submerged in 0.1 M FeSO_4_·7H_2_O solution while being constantly stirred. The pH of the suspension was then brought down to 10~11 by adding 0.1 mol/L NaOH solution dropwise. Following pH correction, the liquid was sealed, agitated for 30 min, and then left alone for 12 h. The composite was collected by suction filtration, dried, and designated as Zn/Fe-BC.

### 2.3. Characterization of Zn/Fe-BC

Surface functional groups in BC, ZnBC, and Zn/Fe-BC were analyzed using a TENSOR27 FT-IR spectrophotometer (Bruker, Jena, Germany), 4000~400 cm^−1^. XPS (Thermo ESCALAB 250 Xi, Waltham, MA, USA) was used to investigate the elemental content, surface chemical composition, and chemical states of these materials. The crystallographic and structural characteristics of the materials were assessed using XRD (D8 Advance; Bruker, Ettlingen, Germany), while their structural ordering was analyzed using Raman spectroscopy (LabRAM HR Evolution; HORIBA, Kyoto, Japan). The sample morphology was investigated utilizing a Sigma 300 Field Emission SEM (ZEISS, Jena, Germany). The specific surface area, pore volume, and pore size distribution of BC, ZnBC, and Zn/Fe-BC were determined using nitrogen adsorption–desorption isotherms via the BET method (Tristar 3020; Micromeritics, Norcross, GA, USA). The zeta potentials of these materials were measured using a PHS-3E analyzer (INESA, Shanghai, China) over a pH range of 2.0~11.0.

### 2.4. Experiments on Cr(VI) Adsorption

In 250 mL conical flasks, static batch tests were carried out with a reciprocating shaker (rotation speed: 150 rpm) with the reaction temperature controlled at 25 °C. The experiments investigated the effects of pH value, dosage, initial concentration, reaction temperature, and coexisting ions on the Cr(VI) removal efficiency of BC, ZnBC, and Zn/Fe-BC. A 1000 mg/L Cr(VI) stock solution was made by dissolving K_2_Cr_2_O_7_ in distilled water, and before use, it was diluted with deionized water. 0.1 mol/L NaOH and HCl were employed to change the initial pH of the solution. The following were the standard experimental conditions: 25 mL of a 50 mg/L Cr(VI) solution was added with 2 g/L of Zn/Fe-BC. Samples were taken at set time intervals; 1 mL of the reaction solution was filtered and immediately analyzed using an ultraviolet spectrophotometer (Shimadzu UV-2450, Kyoto, Japan). Cr(VI) has its highest absorption at 540 nm, and within a certain concentration range, the absorbance shows a linear relationship with the Cr(VI) concentration. The specific detection steps were as follows: (1) After filtering the sample, 1 mL of the supernatant was transferred to a 50 mL colorimetric tube and diluted to the marked scale; (2) 0.5 mL of sulfuric acid-phosphoric acid mixture (1:1, *v*/*v*) was added, followed by 2 mL of chromogenic reagent solution (1,5-diphenylcarbazide solution). The chromogenic reagent was prepared by dissolving 0.2 g of diphenylcarbazide in 50 mL of acetone, then mixing with 50 mL of ultrapure water, and storing it in the dark at 4 °C. The mixture was shaken well; (3) after standing for 5~10 min, the absorbance at 540 nm was measured using a quartz cuvette filled with water as the reference. The following equations were used to determine the equilibrium adsorption capacity (*Q_e_*, mg·g^−1^) and the removal effectiveness (*R*, %):

(1) $$R\left(\%\right)=\frac{\left(C_{i}-C_{e}\right)}{C_{i}}\times 100$$

(2) $$Q_{e}=\frac{\left(C_{i}-C_{e}\right)}{W}\times V$$

Here, *W* is the adsorbent mass (g), *V* the Cr(VI) solution volume, and *C_i_*/*C_e_* the initial/equilibrium Cr(VI) concentrations (mg·L^−1^).

### 2.5. Adsorption Kinetics and Thermodynamics Study

The pseudo-first-order, pseudo-second-order, and Elovich models were used to provide insight into the behavior and mechanism of Cr(VI) adsorption. The particular formulas are as follows [24]:

(3) $$ln\left(Q_{e}-Q_{t}\right)=lnQ_{e}-K_{1}t$$

(4) $$\frac{t}{Q_{t}}=\frac{1}{K_{2}Q_{e}^{2}}+\frac{t}{Q_{e}}$$

(5) $$Q_{t}=\frac{1}{\alpha }ln(\alpha \beta )+\frac{1}{\alpha }lnt$$

The adsorption capability of Cr(VI) at time t is represented by Q_t_ (mg·g^−1^); the rate constants K_1_ (min^−1^) and K_2_ (g·mg^−1^·min^−1^), respectively, represent α and β.

The equation for the Va not Hoff thermodynamic model is used to determine the parameters ΔG^0^ (Equation (6)), K_c_ (Equation (7)), ΔH^0^ (Equation (8)), and ΔS^0^ (Equation (9)). These values show the direction of the reaction and the energy conversion and transfer that takes place throughout this Zn/Fe-BC Cr(VI) adsorption process at different temperatures (288~318 K). The detailed equations are as follows:

(6) $$\Delta G^{0}=-RTln(K_{c})$$

(7) $$K_{c}=55.5\times (\frac{Q_{e}}{C_{e}}\times M_{Cr})$$

(8) $$ln(K_{c})=\frac{-\Delta H^{0}}{RT}+\frac{\Delta S^{0}}{R}$$

(9) $$\Delta G^{0}=-T\Delta S^{0}+\Delta H^{0}$$

where T represents the reaction temperature in Kelvin (K); R is the universal gas constant with a value of 8.314 J·mol^−1^·K^−1^; Δ*G*^0^ denotes the Gibbs free energy; Δ*H*^0^ and Δ*S*^0^ correspond to the changes in enthalpy and entropy, respectively, both measured in kJ·mol^−1^; and K_c_ is the thermodynamic equilibrium constant, which incorporates equilibrium concentration of chromium in solution (C_e_), molar mass of chromium (M_Cr_) and the molar concentration of water in dilute aqueous solutions (55.5 mol·L^−1^).

### 2.6. Isotherm Models

The Freundlich multilayer adsorption model and the Langmuir monolayer adsorption model were used to elucidate the kind and mechanism of Cr(VI) adsorption by Zn/Fe-BC. The specific equations are as follows [25]:

(10) $$Q_{e}=\frac{C_{e}K_{L}Q_{max}}{\left(1+K_{L}C_{e}\right)}$$

(11) $$Q_{e}=K_{F}C_{e}^{1/n}$$

K_L_ (L·mg^−1^) is the affinity constant of the Langmuir model; K_F_ (L·g^−1^) is the Freundlich model constant, and 1/n is the dimensionless empirical constant of the Freundlich model.

## 3. Results and Discussion

### 3.1. Characterization of Biochar

#### 3.1.1. BET Analysis

**Figure 2:**
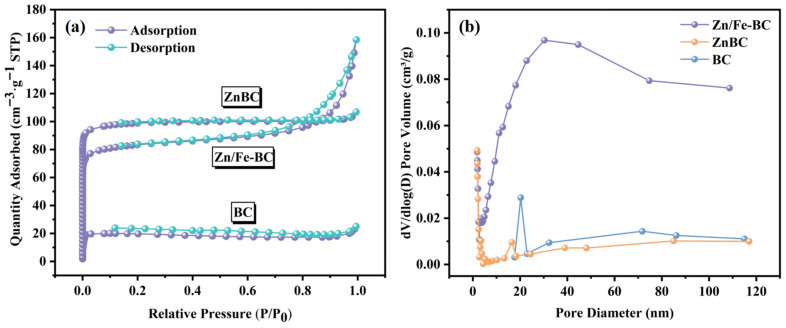
(**a**) Adsorption–desorption isotherms of nitrogen and (**b**) pore size distribution curves of BC, ZnBC, and Zn/Fe-BC.

The N_2_ adsorption–desorption isotherms for BC, ZnBC, and Zn/Fe-BC are displayed in Figure 2a. The isotherms of BC showed typical type I characteristics, indicating that the original biochar was mainly dominated by microporous structure. After ZnCl_2_ activation treatment, the isotherms of ZnBC and Zn/Fe-BC were transformed into type IV, accompanied by an H4 type hysteresis loop, which indicated that the activation treatment successfully introduced abundant mesoporous structure and formed a microporous–mesoporous composite hierarchical pore structure. The ZnCl_2_-activated biochar (ZnBC) exhibited a significantly higher specific surface area (S_BET_ = 375.9 m^2^/g) and total pore volume (*V*_t_ = 0.17 cm^3^/g), which were 4.78 and 4.87 times higher, respectively, than those of raw biochar (BC; S_BET_ = 78.6 m^2^/g and *V*_t_ = 0.03 cm^3^/g). These significant enhancements in surface area and porosity provided additional active sites for Cr(VI) adsorption [26]. After coprecipitation-based magnetization (Fe incorporation into ZnBC via coprecipitation), the resulting Zn/Fe-BC exhibited a slightly smaller specific surface area but a larger pore volume, suggesting partial pore blockage by FeSO_4_·7H_2_O [27]. The results confirmed that the synergistic effect of ZnCl_2_ activation and coprecipitation-based magnetization effectively regulated the pore structure of jujube branch biochar, providing a foundation for its performance improvement.

#### 3.1.2. SEM Analysis

**Figure 3:**
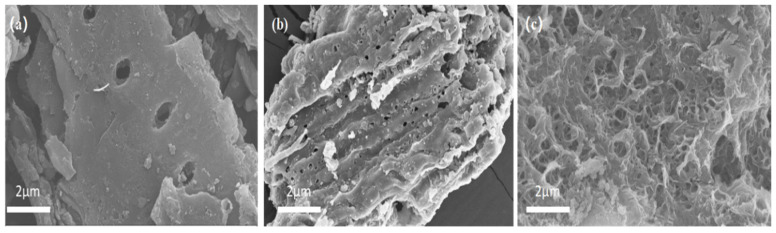
SEM images of (**a**) BC, (**b**) ZnBC, and (**c**) Zn/Fe-BC at 5000× magnification.

The surface structures of BC, ZnBC, and Zn/Fe-BC were analyzed using SEM at a magnification of 5000× (Figure 3). BC exhibited a relatively smooth surface with a limited number of pores (Figure 3a). After ZnCl_2_ activation (Figure 3b), the formation of solid carbon and condensation products during pyrolysis was promoted, the formation of bio-oil was inhibited, and the activation energy of the reaction was increased [28]. In addition, the release of gases such as CO_2_ and H_2_O during pyrolysis etched abundant pore structures on the carbon matrix, thereby increasing the porosity of the resulting ZnBC. Subsequent magnetization produced the Zn/Fe-BC composite (Figure 3c and Figure S2), which exhibited a large number of irregular particles on its surface, indicating the successful dispersion of Fe_3_O_4_ nanoparticles. These fine particles and the more developed pore structure supported the results of the BET analysis.

To further verify that Fe and Zn are effectively incorporated into the composite rather than simply adsorbed without counterions, we conducted EDS analysis (Figure S1). Zn was detected in ZnBC (Figure S1b) with an atomic percentage of 0.34%, confirming the successful introduction of Zn during activation and the absence of obvious interfering counterions. Both Fe (7.87%) and Zn (0.38%) were detected in Zn/Fe-BC (Figure S1c). The presence of these elements and the absence of significant counterion peaks indicate that Fe and Zn were effectively incorporated into the composite structure, rather than merely adsorbed on the surface without stable bonding.

#### 3.1.3. XRD and Raman Spectroscopy Analyses

**Figure 4:**
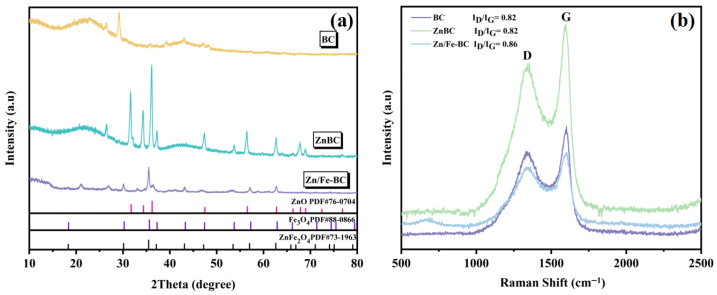
BC, ZnBC, and Zn/Fe-BC XRD patterns and Raman spectra (**a**,**b**).

The XRD patterns of BC, ZnBC, and Zn/Fe-BC are shown in Figure 4a. BC exhibited two broad peaks in the 2*θ* ranges of 15–30° and 35–45°, which were attributed to amorphous carbon, and a diffraction peak at 2*θ* = 29.2°, which corresponded to SiO_2_. While displaying distinct diffraction peaks at 2*θ* = 31.7°, 34.4°, 36.2°, 47.5°, 56.5°, 62.7°, 66.3°, 67.8°, 69.0°, and 72.5°, ZnBC maintained the amorphous carbon peaks. These peaks verified the development of the ZnO crystalline phase by matching the (100), (002), (101), (102), (110), (103), (200), (112), (201), and (004) planes of ZnO (PDF#76-0704). Zn/Fe-BC displayed new diffraction peaks at 2*θ* = 18.3°, 30.1°, 35.5°, 43.1°, 53.5°, 57.0°, and 62.7°. These peaks were assigned to the (111), (220), (311), (400), (422), (511), and (440) planes, respectively, matching the standard patterns for Fe_3_O_4_ (PDF#88-0866) and ZnFe_2_O_4_ (PDF#73-1963) [29]. These findings confirmed that Fe was successfully incorporated in the form of Fe_3_O_4_ and that the ZnFe_2_O_4_ composite was formed [30]. The crystalline phases provided essential structural features for the adsorption performance of the composite.

Raman spectroscopy was employed to analyze the graphitization degree and structural defects of BC, ZnBC, and Zn/Fe-BC (Figure 4b). All three materials exhibited characteristic peaks of D and G bands [31]. The E_2g_ vibrational mode of sp^2^-hybridized graphitic carbon was represented by the G band, whereas the D band was associated with disordered carbon and structural flaws [32]. The I_D_/I_G_ ratios for BC and ZnBC were both 0.82, slightly lower than that of Zn/Fe-BC (0.86), indicating that Zn/Fe-BC possessed more structural defects and a lower degree of graphitization. These defects provided abundant active sites and enhanced surface reactivity, thus promoting Cr(VI) adsorption [33]. Furthermore, the graphitic domains retained in the composite facilitated electron transfer, further improving Cr(VI) removal efficiency.

#### 3.1.4. FT-IR and XPS Analyses

**Figure 5:**
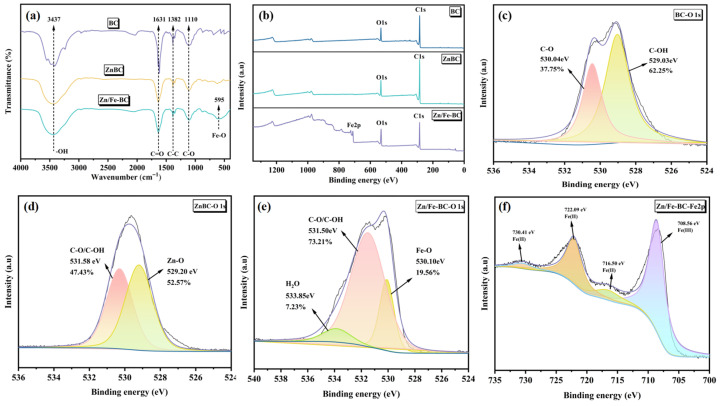
(**a**) FT-IR spectra of BC, ZnBC, and Zn/Fe-BC. (**b**) XPS survey spectra and (**c**–**e**) high-resolution O 1s spectra. (**f**) The Zn/Fe-BC Fe 2p spectrum.

The FT-IR spectra of BC, ZnBC, and Zn/Fe-BC are shown in Figure 5a. The broad absorption peak at 3437 cm^−1^ was found to be caused by the stretching vibration of -OH, while the peaks at 1631, 1382, and 1100 cm^−1^ were found to be caused by the stretching vibrations of C=O, C-C, and C-O, respectively. According to these findings, the Zn/Fe-BC surface has functional groups that include oxygen [34]. The peak detected at 586 cm^−1^ corresponded to Fe-O bonds, verifying the successful integration of Fe_3_O_4_ into Zn/Fe-BC.

Compared to BC and ZnBC, Zn/Fe-BC demonstrated a clear Fe 2p binding energy peak (Figure 5b). Specifically, the high-resolution Fe 2p spectrum of Zn/Fe-BC (Figure 5f) showed distinct peaks at 708.56 and 722.09 eV, corresponding to Fe(III) and Fe(II), respectively, supporting the formation of Fe_3_O_4_ as indicated by XRD results [35]. The O1s spectra before and after modification (Figure 5c–e) revealed three deconvoluted peaks at 530.10, 531.50, and 533.85 eV in Zn/Fe-BC, assigned, respectively, to oxides Fe-O, C-O/OH, and H_2_O as their characteristic peaks. Notably, ZnBC displayed a distinct Zn-O peak in its O1s spectrum. The addition of Zn and Fe improved the production of functional groups containing oxygen, as evidenced by the rise in the relative content of C-OH in Zn/Fe-BC from 62.25% to 73.21%. This enhancement could be attributed to the interaction or redox reaction between metal oxides and the biochar surface [36], which improved the hydrophilicity and surface reactivity of Zn/Fe-BC. The appearance of the Fe-O peak after modification further confirmed the formation of iron oxides, consistent with FT-IR findings.

### 3.2. Cr(VI) Adsorption Experiments

#### 3.2.1. Effects of Adsorbent Dosage

**Figure 6:**
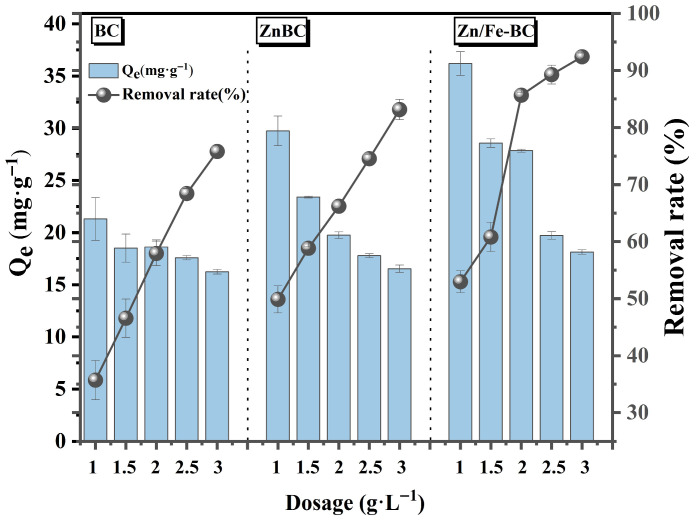
Cr(VI) removal efficiencies and adsorption capacities of BC, ZnBC, and Zn/Fe-BC at different dosages.

The adsorption capacities and removal efficiencies of Cr(VI) for BC, ZnBC, and Zn/Fe-BC at varying doses are displayed in Figure 6. Compared to BC and ZnBC, Zn/Fe-BC showed significantly higher Cr(VI) removal efficiency and adsorption capacity across all dosages. At a dosage of 2 g/L, the Cr(VI) removal efficiency of Zn/Fe-BC reached 85.66%, significantly higher than those of BC (57.93%) and ZnBC (66.21%). Similarly, the adsorption capacity of Zn/Fe-BC (27.85 mg/g) was substantially higher than that of BC (18.61 mg/g) and ZnBC (19.75 mg/g). These improvements were contributed by the large S_BET_ (269.32 m^2^/g) and *V*_t_ (0.2414 cm^3^/g) of Zn/Fe-BC (Table 1), as well as its high defect density and abundant surface functional groups [37]. Increasing the dosage of Zn/Fe-BC from 2 to 3 g/L led to a notable improvement in Cr(VI) removal efficiency, although the adsorption capacity declined. This phenomenon may be ascribed to the aggregation or overlapping of active sites under higher dosages, which narrowed the effective contact area and extended the diffusion path of Cr(VI) [38]. Nevertheless, the large S_BET_ and abundant adsorption sites in Zn/Fe-BC maintained the likelihood of contact with Cr(VI) [39]. Thus, in order to reconcile high Cr(VI) removal efficiency with effective adsorption capacity, 2 g/L was determined to be the ideal dose for Zn/Fe-BC.

#### 3.2.2. Effects of the Solution pH

**Figure 7:**
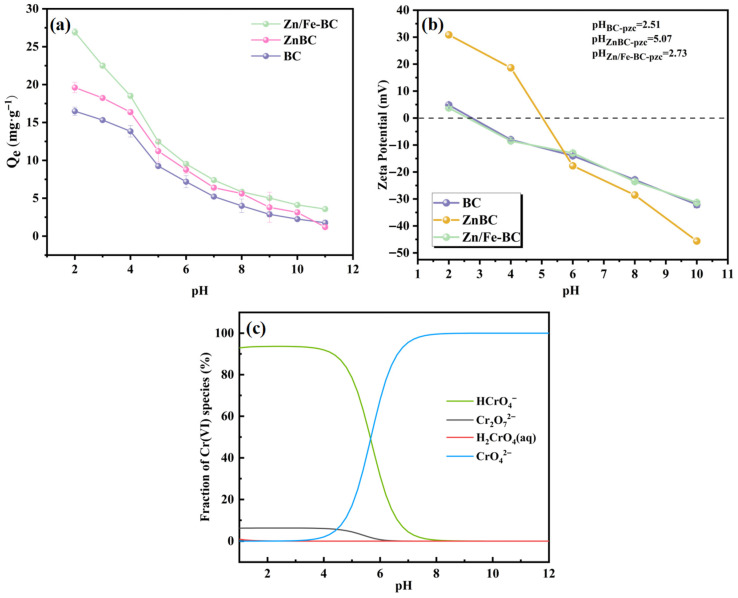
(**a**) Effects of the solution pH on the Cr(VI) removal efficiencies of BC, ZnBC, and Zn/Fe-BC. (**b**) Zeta potential curves and pH_pzc_ values of BC, ZnBC, and Zn/Fe-BC. (**c**) Distribution of Cr(VI) species with variations in pH.

The pH of the solution affects the surface charge distribution of BC, ZnBC, and Zn/Fe-BC, as seen in Figure 7c. In aqueous environments, Cr(VI) can exist in the forms of H_2_CrO_4_, HCrO_4_^−^ CrO_4_^2−^, and Cr_2_O_7_^2−^, depending on the pH. The impact of the solution pH (2.0–11.0) on the Cr(VI) removal efficiencies of BC, ZnBC, and Zn/Fe-BC is shown in Figure 7a. At pH 2, Zn/Fe-BC demonstrated the highest Cr(VI) adsorption capacity, which reached 27.25 mg/g. The adsorption capacity of Zn/Fe-BC, however, dropped precipitously with increasing pH and stayed low at alkaline conditions. The point of zero charge (pH_pzc_) values for BC, ZnBC, and Zn/Fe-BC were 2.51, 5.07, and 2.73, respectively (Figure 7b). The higher pH_pzc_ of ZnBC compared to that of BC could be attributed to the presence of polar Zn-O bonds formed via chemical bonding of Zn^2+^ after ZnCl_2_ activation. Due to the higher electronegativity of Zn relative to the carbon matrix, the surface charge density of ZnBC increased significantly. Moreover, the Zn-O bonds exhibited a strong affinity for protons and were easily protonated under acidic conditions. The reduced pH_PZC_ of Zn/Fe-BC could be due to the introduction of iron-based sites with inherently low pH_PZC_ values, along with the partial neutralization of positive surface charges due to physical coverage. At pH < 2.73, the surface of Zn/Fe-BC was highly protonated due to H^+^ accumulation, resulting in a positively charged surface. This protonation promoted the reduction and ion exchange interactions involving Fe^2+^ and the active sites of Zn/Fe-BC, thereby enhancing Cr(VI) removal efficiency [40]. In contrast, when the pH exceeded 2.73, the surface of Zn/Fe-BC became negatively charged and repelled anionic Cr(VI) species. Furthermore, as the pH increased, the concentration of OH-in the solution rose as well, which led to competing adsorption with Cr(VI) species (such as HCrO_4_^−^, CrO_4_^2−^, and Cr_2_O_7_^2−^) and a decrease in the removal efficiency of Cr(VI) [41].

#### 3.2.3. Coexisting Ion Effects

**Figure 8:**
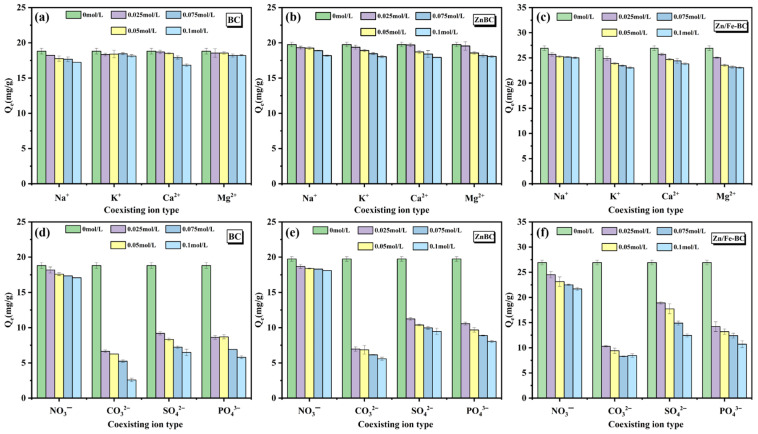
Effects of coexisting ions on the Cr(VI) removal efficiencies of BC, ZnBC, and Zn/Fe-BC: (**a**–**c**) interfering cations and (**d**–**f**) interfering anions.

Figure 8a–f show the effects of four anions (SO_4_^2−^, NO_3_^−^, PO_4_^3−^, and CO_3_^2−^) and four cations (Na^+^, Ca^2+^, K^+^, and Mg^2+^) on the Cr(VI) removal efficiencies of BC, ZnBC, and Zn/Fe-BC under competitive adsorption conditions (initial solution pH adjusted to 2.0, without using buffer solutions during the experiment). Compared with the cations, the anions exhibited a stronger inhibitory effect on Cr(VI) removal by the three adsorbents. Among the anions, CO_3_^2−^ showed the most significant inhibition (Figure 8d–f) [42]. At CO_3_^2−^ oncentrations ranging from 0.025 to 0.1 mol/L, the Cr(VI) removal efficiency of Zn/Fe-BC decreased by 20.36~22.69%. This decrease may be explained by the hydrolysis of CO_3_^2−^, which raises the pH of the solution and decreases the protonation degree of HCrO_4_^−^, thus decreasing its electrostatic attraction to the positively charged sites on the adsorbent surface. For cations (Figure 8a–c), the inhibitory effects followed the order of Mg^2+^ > Ca^2+^ > K^+^ > Na^+^ across the concentration range of 0.025~0.1 mol/L. When 0.1 mol/L of Mg^2+^ or Ca^2+^ was present, the Cr(VI) adsorption capacity of Zn/Fe-BC decreased by 7.51% or 5.91%, respectively. This could be due to the higher charge densities of Mg^2+^ and Ca^2+^ compared to those of K^+^ and Na^+^ [43], making them more likely to bind to negatively charged functional groups (such as -COO^−^) on the Zn/Fe-BC surface, thereby competing with Cr(VI) species (CrO_4_^2−^/HCrO_4_^−^) for adsorption sites [44]. Overall, the inhibitory effect of anions on Cr(VI) removal is significantly stronger than that of cations, which is attributed to both the competition for adsorption sites and the slight pH drift caused by anions.

### 3.3. Adsorption Characteristics of Cr(VI) by BC, ZnBC, and Zn/Fe-BC

#### 3.3.1. Adsorption Kinetics

**Figure 9:**
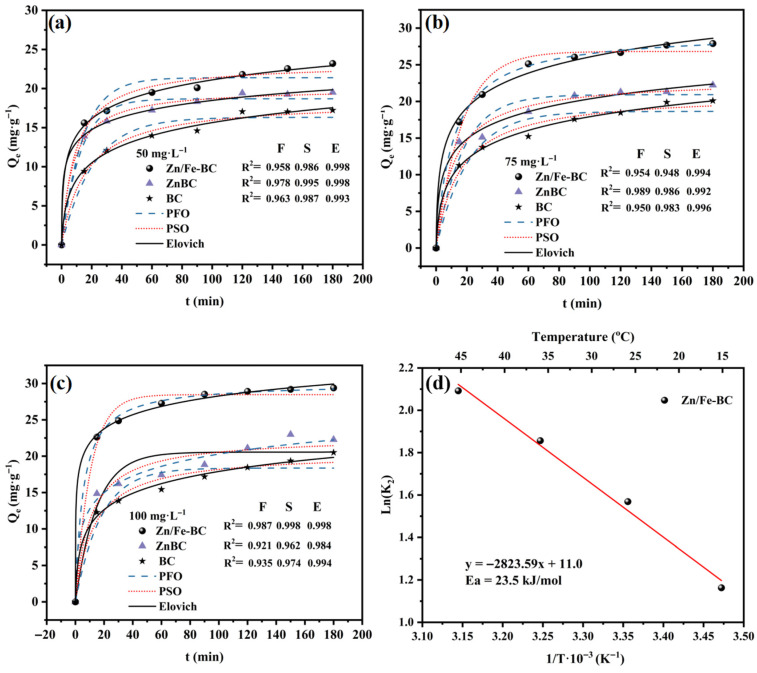
Kinetic model fitting for Cr(VI) adsorption on BC, ZnBC, and Zn/Fe-BC using (**a**–**c**) kinetic (PFO, PSO, and Elovich) models; (**d**) Arrhenius plot for activation energy calculation of Zn/Fe-BC.

The results of the kinetic fitting for Cr(VI) adsorption using BC, ZnBC, and Zn/Fe-BC are shown in Figure 9. The Cr(VI) adsorption process consists of three distinct phases: a rapid adsorption phase, a slower adsorption phase, and a final equilibrium phase. During the 0~30 min, Cr(VI) adsorption followed a free diffusion mechanism, as the plentiful surface active sites enabled rapid adsorption of Cr(VI). Between 30 and 120 min, chemical adsorption became the dominant mechanism, with the gradual occupation of active sites reducing the adsorption rate, approaching equilibrium [45]. In the final stage (120~180 min), the process reached dynamic equilibrium, with Cr(VI) adsorption occurring via chemical adsorption or multilayer diffusion. At low initial Cr(VI) concentrations, adsorption mainly occurred on the biochar surface. However, at higher concentrations, Cr(VI) ions could penetrate the internal pore structure, allowing adsorption at internal and external sites [46].

The kinetics of Cr(VI) adsorption onto Zn/Fe-BC were investigated using the PFO, PSO, and Elovich models. Comparison of the fitting results (Table S1) revealed that the PSO model demonstrated the best fit, with correlation coefficients (*R*^2^) closer to 1 across all tested concentrations. Specifically, at Cr(VI) concentrations of 50, 75, and 100 mg/L, the *R*^2^ values of BC, ZnBC, and Zn/Fe-BC were 0.975~0.996, 0.954~0.995, and 0.984~0.998, respectively. Moreover, the theoretical equilibrium adsorption capacities (Q_e_) obtained from the PSO model were highly consistent with the experimental values (Q_exp_). In particular, the Zn/Fe-BC Q_exp_ values were 23.20, 27.08, and 29.87 mg/g for the starting Cr(VI) concentrations of 50, 75, and 100 mg/L, respectively. These values were consistent with the Q_e_ values predicted by the PSO model (23.27, 26.92, and 30.09 mg/g) and more accurate than those obtained from the PFO model (21.38, 26.61, and 28.47 mg/g). The Elovich model also showed high *R*^2^ values (0.992–0.998, Table S1), indicating that Zn/Fe-BC had a heterogeneous surface with variations in energy distribution at adsorption sites. This suggested that Cr(VI) removal involved surface electron transfer or valence bonding [47]. Overall, these kinetic analysis results confirmed that Cr(VI) adsorption by Zn/Fe-BC was mainly governed by chemisorption, involving non-uniform surface active sites.

Moreover, the activation energy *E_a_* for Cr(VI) adsorption on Zn/Fe-BC, calculated via the Arrhenius equation using *K*_2_ at different temperatures, was approximately 23.5 kJ/mol (Figure 9d). Falling within the 20–40 kJ/mol range, this value further confirms chemisorption-dominated adsorption, consistent with kinetic model fitting results.

#### 3.3.2. Adsorption Thermodynamics

**Figure 10:**
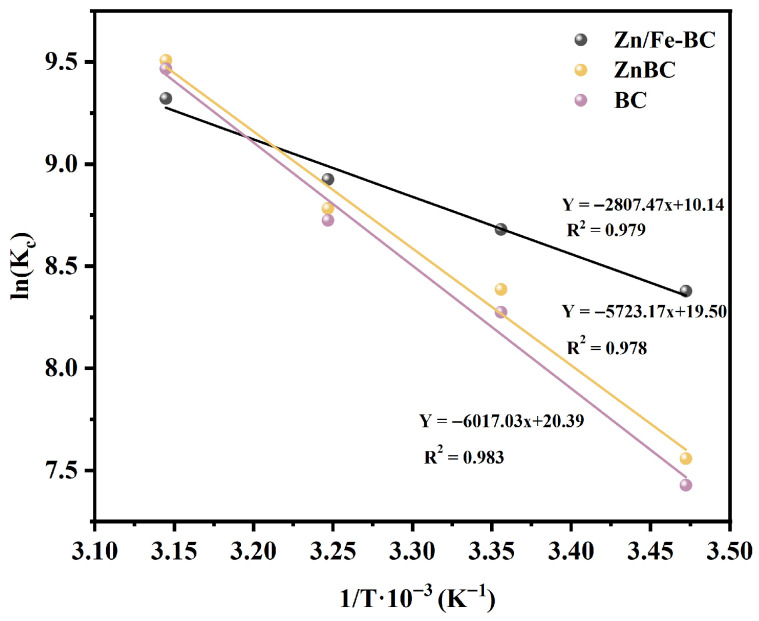
Thermodynamic model fitting for the adsorption of Cr(VI) onto BC, ZnBC, and Zn/Fe-BC.

The thermodynamic behavior of Cr(VI) adsorption onto BC, ZnBC, and Zn/Fe-BC was evaluated in the temperature range of 288–318 K, as depicted in Figure 10 and Table S2. As shown in Figure 10, the linear fitting of lnK_c_ versus 1/T for each material yielded good linear relationships, with *R*^2^ values of 0.979 for Zn/Fe-BC, 0.978 for ZnBC, and 0.983 for BC, indicating the reliability of the thermodynamic calculation results.

The standard enthalpy changes (Δ*H*^0^) for BC and ZnBC were 50.03 kJ/mol and 47.583 kJ/mol, respectively, both of which were considerably greater than the value for Zn/Fe-BC, which was 23.34 kJ/mol. The higher Δ*H*^0^ values for BC and ZnBC suggested that these materials required a greater amount of energy to facilitate Cr(VI) adsorption [48]. In contrast, the lower Δ*H*^0^ for Zn/Fe-BC indicated a moderate interaction strength, suggesting a synergistic mechanism involving physical and chemical adsorption. The standard Gibbs free energy changes (Δ*G*^0^) for Zn/Fe-BC were negative, ranging from −19.83 to −24.69 kJ/mol, indicating that the adsorption of Cr(VI) onto Zn/Fe-BC was a spontaneous process [49]. An increase in temperature not only enhanced the affinity between Zn/Fe-BC and Cr(VI) but also facilitated the activation of surface adsorption sites. The standard entropy change (Δ*S*^0^) of Zn/Fe-BC was 84.28 J/mol·K, which was significantly lower than those of BC (169.54 J/mol·K) and ZnBC (162.17 J/mol·K), suggesting that Cr(VI) adsorption on Zn/Fe-BC resulted in a smaller increase in system disorder and involved higher selectivity at the adsorption sites [50].

#### 3.3.3. Adsorption Isotherms

**Figure 11:**
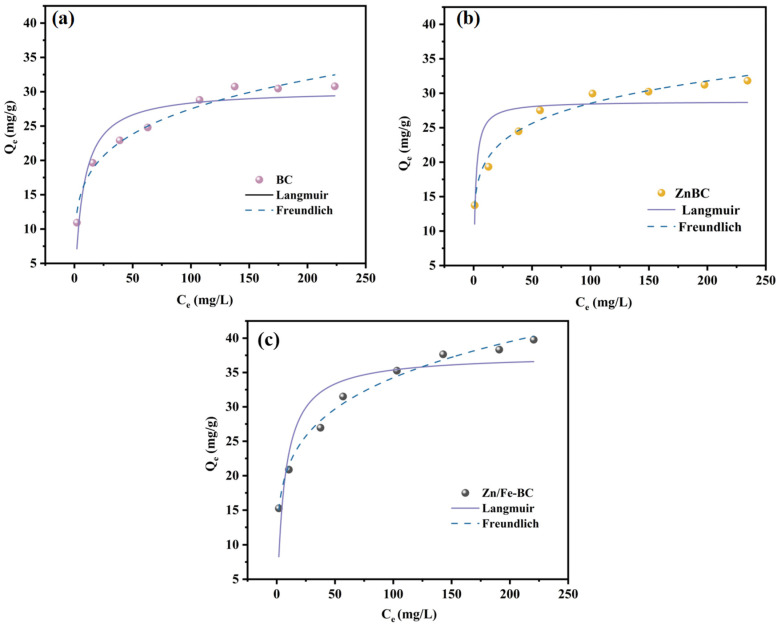
Adsorption isotherms onto (**a**) BC, (**b**) ZnBC, and (**c**) Zn/Fe-BC.

The adsorption isotherms of BC and Zn/Fe-BC are shown in Figure 11 and Table 2. At low initial Cr(VI) concentrations, the surfaces of both adsorbents contained abundant active sites and functional groups. A larger adsorption capacity resulted from a greater chance of Cr(VI) coming into contact with accessible adsorption sites when the initial concentration of Cr(VI) rose. However, the Cr(VI) adsorption rate dropped and ultimately reached dynamic equilibrium when these active sites grew saturated and functional groups were exhausted. The adsorption behavior was evaluated using the Langmuir (uniform surface) and Freundlich (non-uniform surface) isotherm models. For both BC and Zn/Fe-BC, the *R*^2^ values of the Freundlich model (0.969 and 0.990, respectively) were greater than the corresponding values from the Langmuir model (0.870 and 0.786). These results suggested that Cr(VI) adsorption on BC and Zn/Fe-BC occurred on heterogeneous surfaces and conformed to the multilayer adsorption mechanism. Furthermore, the saturated adsorption capacity (Q_m_) of Zn/Fe-BC reaches 37.658 mg/g, a value significantly superior to the Cr(VI) removal performance of some magnetic nano-adsorbents listed in Table S4.

#### 3.3.4. Cr(VI) Removal Mechanisms of Zn/Fe-BC

The removal of Cr(VI) by Zn/Fe-BC was found to involve a number of mechanisms, including reduction, electrostatic interaction, precipitation, complexation, and pore filling, based on the findings of characterization analyses and batch adsorption examinations.

**Figure 12:**
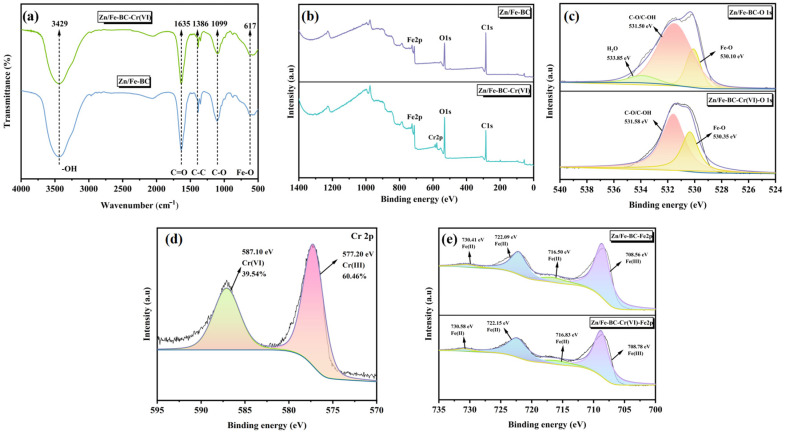
Zn/Fe-BC FT-IR and XPS spectra before and following Cr(IV) adsorption (**a**) FT-IR spectra; (**b**) XPS survey spectra; (**c**) O 1s; (**d**) Cr 2p; (**e**) Fe 2p.

FT-IR spectra collected prior to and following Cr(VI) adsorption (Figure 12a) were analyzed, revealing changes in the surface functional groups of Zn/Fe-BC. The characteristic peaks of Zn/Fe-BC were weakened after the adsorption, confirming that the active functional groups on Zn/Fe-BC interacted with Cr(VI). To further elucidate the adsorption mechanism, XPS was used to analyze changes in the surface chemical composition of Zn/Fe-BC before and after Cr(VI) adsorption. After adsorption, a new Cr 2p peak appeared in the XPS survey spectrum (Figure 12b), demonstrating that Zn/Fe-BC had successfully captured Cr(VI) [51]. In the high-resolution spectrum of Cr 2p (Figure 12e), two obvious characteristic peaks were detected: the peak at the binding energy of 587.10 eV corresponded to Cr(VI), and the peak at 577.20 eV was the typical characteristic of Cr(III), which confirmed that Cr(VI) had an obvious reduction transformation after contacting with the material. It is worth noting that XPS is a surface-sensitive technique, and the quantitative partitioning of Cr between solution and adsorbent surface was not directly measured in this study. The high-resolution O1s spectrum (Figure 12c) revealed a reduction in the C-O/C-OH peak and the disappearance of the H_2_O peak after adsorption, as well as an increase in the Fe-O peak intensity from 19.56% to 34.79%. These changes suggested that C-O/C-OH coordinated with Cr(VI), while H_2_O contributed protons to facilitate the redox reaction, resulting in Fe-O formation [52]. This finding was consistent with FT-IR results, where the intensities of -OH and C-O peaks decreased after Cr(VI) adsorption.

In the Fe 2p spectrum (Figure 12d), Fe(II) was identified by the peaks at 730.41, 722.09, and 716.50 eV, while Fe(III) was identified by the peak at 708.56 eV. The Fe(II) peaks were weaker following Cr(VI) adsorption, [53], suggesting that Fe(II) contributed electrons to convert Cr(VI) to Cr(III), as shown in Equations (12)–(15). The produced Cr(III) then formed stable precipitates with OH^−^ and Fe(III), as shown in Equations (16) and (17). The distribution of elements after the reaction, as depicted in Figure S2, provides further evidence for the formation of Cr(III)-related precipitates. Figure S2 illustrates the contents of total Cr(T), Cr(VI), Cr(III), Fe, and Zn post-reaction. The significantly higher content of Cr(T) relative to Cr(VI) suggests that a substantial fraction of Cr(VI) underwent reduction to Cr(III). Simultaneously, the detectable concentrations of Fe and Zn also reflect their participation in the redox, complexation, and precipitation processes, in line with the aforementioned mechanisms.

(12) $${Cr}_{2}O_{7}^{2-}+H_{2}O\to {2HCrO}_{4}^{-},$$

(13) $${3Fe}^{2+}+2HCrO_{4}^{-}+7H^{+}\to {3Fe}^{3+}+{Cr}^{3+}+{4H}_{2}O,$$

(14) $$CrO_{4}^{2-}+{2Fe}^{2+}+{4H}_{2}O\to {Cr}^{3+}+{3Fe}^{3+}+{8OH}^{-},$$

(15) $${Cr}^{3+}+3OH^{-}\to {Cr(OH)}_{3}↓,$$

(16) $${nCr}^{3+}+{\left(1-n\right)Fe}^{3+}+{3H}_{2}O⇌{Cr}_{n}{Fe}_{\left(1-n\right)}{\left(OH\right)}_{3}↓+{3H}^{+},$$

(17) $${nCr}^{3+}+{\left(1-n\right)Fe}^{3+}+{2H}_{2}O⇌{Cr}_{n}{Fe}_{\left(1-n\right)}OOH↓+{3H}^{+},$$

**Figure 13:**
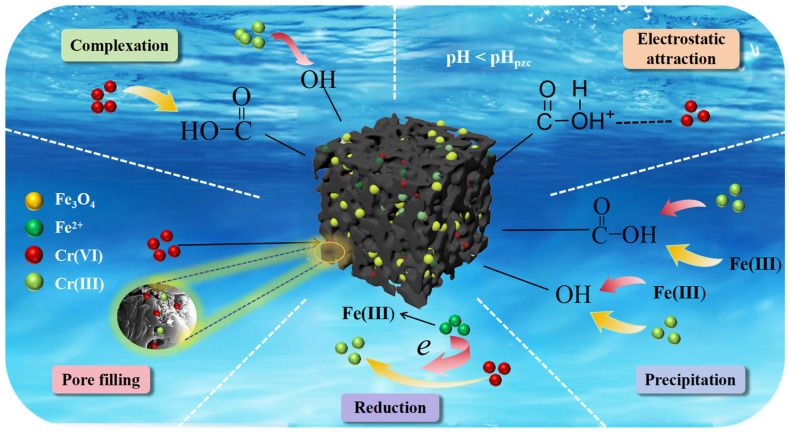
Cr(VI) removal mechanisms of Zn/Fe-BC.

In summary, the removal of Cr(VI) by Zn/Fe-BC involved several synergistic mechanisms, including reduction, complexation, and precipitation. Protonation caused the Zn/Fe-BC surface to become positively charged in an acidic environment, which made it easier to remove Cr(VI) and Cr(III) by ion exchange and electrostatic attraction. In addition, the porous structure and high specific surface area of Zn/Fe-BC supported pore filling as a fundamental mechanism for Cr(VI) adsorption (Figure 13).

#### 3.3.5. Reusability Analysis

**Figure 14:**
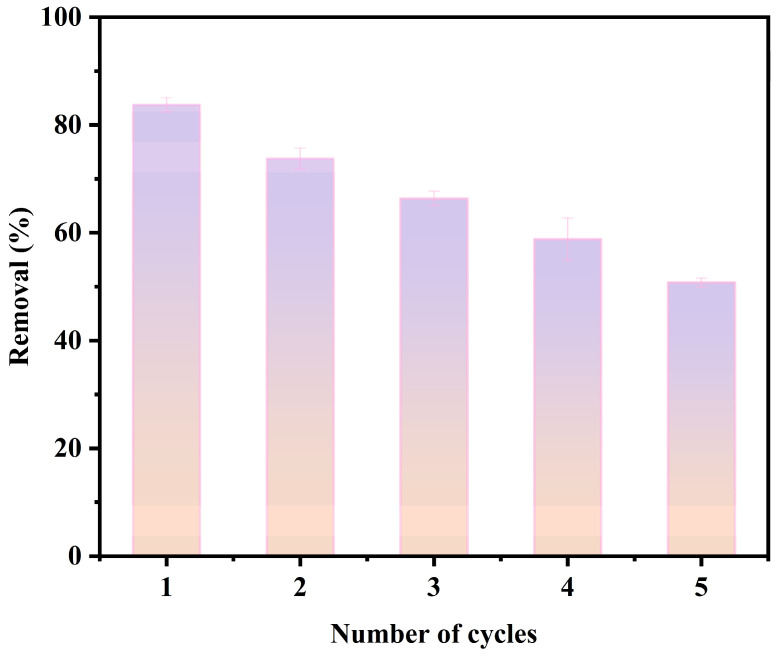
Reusability of Zn/Fe-BC.

The cyclic regeneration performance of Zn/Fe-BC is illustrated in Figure 14. Following five adsorption–desorption cycles, the Cr(VI) desorption efficiency of Zn/Fe-BC remained above 60.35%, indicating that the material maintained a high adsorption performance even after multiple cycles. In order to provide a highly alkaline environment, a 0.1 M NaOH solution was used as the desorption agent. This allowed OH^−^ ions to compete with CrO_4_^2−^ and Cr_2_O_7_^2−^ for active binding sites on the Zn/Fe-BC surface, displacing them by ion exchange. However, iron leaching at high pH values had a negative impact on the desorption efficiency. In particular, Fe(II) and NaOH interacted to generate Fe(OH)_2_, which led to the loss of active adsorption sites and the creation of persistent complexes that prevented Cr(VI) from being read-absorbed in later cycles. Thus, the notable reduction in Cr(VI) removal efficiency was due to pore obstruction or the depletion of active sites on the Zn/Fe-BC surface. This finding also reflected that Cr(VI) was firmly adsorbed on Zn/Fe-BC, presenting a challenge for complete regeneration.

To address these regeneration challenges, three targeted optimization strategies are proposed: (1) Optimize the desorbent system by replacing the single 0.1 M NaOH with a mixed system of 0.05 M NaOH + 0.5 M NaCl. The Cl^−^ in NaCl can form stable [FeCl_4_]^2−^ complexes with Fe(II), reducing Fe(OH)_2_ formation and iron leaching, while low-concentration NaOH ensures effective competitive desorption of Cr(VI) by OH^−^. (2) Introduce ultrasonic-assisted desorption (40 kHz, 200 W, 15 min) during the process; the cavitation effect of ultrasound accelerates desorbent diffusion into pores, promoting the removal of residual Cr(VI) and Fe(OH)_2_ complexes to alleviate pore blockage. (3) Implement post-desorption material repair: immerse the desorbed Zn/Fe-BC in 0.01 M FeSO_4_ solution for 30 min to replenish lost Fe(II) active sites via ion exchange and then roast it at 300 °C for 1 h under N_2_ atmosphere to stabilize the replenished Fe(II) on the biochar surface.

## 4. Conclusions

In this study, Zn/Fe-BC was prepared from jujube branches via an impregnation pyrolysis–coprecipitation method. The efficiency of Zn/Fe-BC for the removal of Cr(VI) from wastewater was validated by characterization tests (SEM, BET, XRD, Raman, XPS, and FT-IR) as well as adsorption studies. ZnCl_2_ promoted the Cr(VI) removal by the adjustment of the microstructure of the material, while Fe ions promoted the Cr(VI) reduction and increased the recyclability of the material. Zn/Fe-BC presented significant pH dependence for Cr(VI) removal, with its best performance achieved near pH ≈ 2, and for real-water treatment, this meant pre-conditioning the water to reach pH ≈ 2; at this pH, Zn/Fe-BC achieved its maximum adsorption capacity of 27.85 mg/g within 180 min. Coexisting anions had an impact on Cr(VI) in the following order: CO_3_^2−^ > PO_4_^3−^ > SO_4_^2−^ > NO_3_^−^. The adsorption was endothermic and spontaneous, and the data followed the Freundlich isotherm with pseudo-second-order kinetics. Cr(VI) removal by Zn/Fe-BC was driven by physical adsorption and chemical reduction, involving a synergistic combination of pore filling, electrostatic adsorption, reduction, complexation, and precipitation. Zn leaching from Zn/Fe-BC was near regulatory thresholds; mitigation measures such as polymer coating or secondary roasting may be needed for practical use. This study presents a novel, environmentally friendly, and effective strategy for Cr(VI) remediation, offering a valuable reference for environmental protection efforts.

## Figures and Tables

**Table 1:** BC, ZnBC, and Zn/Fe-BC surface area and pore structure characteristics.

Sample	S_BET_ (m^2^/g)	V_t_ (cm^3^/g)	V_micro_ (cm^3^/g)	D_P_ (nm)
BC	78.6	0.04	0.03	1.89
ZnBC	375.9	0.17	0.14	1.63
Zn/Fe-BC	269.3	0.24	0.11	1.90

**Table 2:** Model parameters for the adsorption of Cr(VI) onto BC, ZnBC, and Zn/Fe-BC adsorption.

Sample	Langmuir			Freundlich		
	Q_m_/(mg/g)	K_L_	R^2^	K_F_	1/n	R^2^
BC	30.312	0.144	0.870	10.62	0.207	0.969
ZnBC	28.829	0.727	0.660	13.95	0.155	0.974
Zn/Fe-BC	37.658	0.154	0.786	13.26	0.206	0.990

## Data Availability

The data can be found in the article and its Supplementary Materials.

## Supplementary Materials

The following supporting information can be downloaded at: https://www.mdpi.com/article/10.3390/nano15201586/s1, Table S1: Kinetic model parameters; Table S2: Thermodynamic parameters; Table S3: Jujube branch chemical composition; Table S4: Adsorption capacities: Zn/Fe-BC vs. other biochars; Table S5: Determination results of total carbon (TC), inorganic carbon (IC), and total organic carbon (TOC) in different solutions; Table S6: Comparison of preparation costs of different Cr(VI) removal adsorbents; Figure S1: SEM image, element distribution, and EDS spectrum of (a) BC, (b) ZnBC, and (c) Zn/Fe-BC; Figure S2: Distribution of element contents after reaction; Figure S3: UV–Vis absorption spectra of Cr(VI) stock solution and solution after reaction; Figure S4: SEM images of Zn/Fe-BC. References [54,55,56,57,58,59,60,61,62] are cited in the supplymentary materials.
