# Supplementary material for: Preparation of ZnCl2-Activated Magnetic Biochar and Its Performance in Removing Hexavalent Chromium from Water
Source: Nanomaterials (Basel). 2025 Oct 17;15(20):1586. doi: 10.3390/nano15201586 (PMC12566338; doi:10.3390/nano15201586)
Supplement: Supplementary file 1 [file nanomaterials-15-01586-s001.zip › nanomaterials-3864703-supplementary.pdf]

Supporting Information

Preparation of $ZnCl_2$-activated magnetic biochar and its performance in removing hexavalent chromium from water

Pingqiang Gao [1,2], Zhe Tan [1], Yonghao Yan [1], Min Yang [1], Shuai Han [1], Chen Yang [1], Shuai Li [1], Yan Zhang [1,*]

[1] School of Chemistry and Chemical Engineering, Yulin University, No. 51 Chongwen Road, Yulin City, Shaanxi, 719000, P. R.China

[2] Yulin Engineering Research Center of Coal Chemical Wastewater, Yulin University, No. 51 Chongwen Road, Yulin 719000, China

* Corresponding author: zhangyan@yulinu.edu.cn (Y.Z.);

Tel:+86-0912-3282878. E-mail address: zhangyan@yulinu.edu.cn (Y. Z).

Table S1. Kinetic model parameters.

Models and parameters	50 mg·L^{-1}			75 mg·L^{-1}			100 mg·L^{-1}		
	BC	ZnBC	Zn/Fe-BC	BC	ZnBC	Zn/Fe-BC	BC	ZnBC	Zn/Fe-BC
Pseudo-first order									
k_1/(mg·g^{-1}·min^{-1})x10^{-2}	0.047	0.079	0.071	0.048	0.058	0.060	0.056	0.067	0.095
Q_t/(mg·g^{-1})	16.32	18.68	21.38	18.64	20.92	26.61	18.36	20.55	28.47
R_1^2	0.963	0.978	0.958	0.950	0.954	0.989	0.935	0.921	0.987
Pseudo-second order									
k_2/(g·mg^{-1}·min^{-1})x10^{-2}	0.0034	0.0068	0.0048	0.0031	0.0038	0.0032	0.0038	0.0043	0.0062
Q_t/(mg·g^{-1})	18.46	20.07	23.27	21.06	23.02	26.92	20.49	22.66	30.09
R_2^2	0.987	0.996	0.986	0.983	0.983	0.948	0.974	0.962	0.998
Elovich									
α/(mg·g^{-1}·min^{-1})	3.91	65.72	28.44	4.69	12.86	17.65	7.43	15.19	664.06
β/(mg·g^{-1})	0.31	0.43	0.32	0.27	0.29	0.23	0.30	0.30	0.35
R_3^2	0.993	0.995	0.998	0.996	0.992	0.994	0.994	0.984	0.998

Table S2. Thermodynamic parameters.

Temperature		BC	ZnBC	Zn/Fe-BC
288	ΔG (kJ/mol)	-17.78	-18.09	-19.83
	ΔH (kJ/mol)	50.026	47.583	23.341
	ΔS (J/mol·K)	169.538	162.173	84.281
298	ΔG (kJ/mol)	-20.50	-20.78	-21.69
	ΔH (kJ/mol)	50.026	47.583	23.341
	ΔS (J/mol·K)	169.538	162.173	84.281
308	ΔG (kJ/mol)	-22.34	-22.49	-22.87
	ΔH (kJ/mol)	50.026	47.583	23.341
	ΔS (J/mol·K)	169.538	162.173	84.281
318	ΔG (kJ/mol)	-25.03	-25.13	-24.69
	ΔH (kJ/mol)	50.026	47.583	23.341
	ΔS (J/mol·K)	169.538	162.173	84.281

Jujube branches were collected from Jiaxian, Yulin, China. The samples were thoroughly rinsed with deionized water to remove surface impurities, then dried in an oven at 105°C to constant weight. After drying, the branches were crushed using a grinder and sieved through a 150-mesh screen to obtain a uniform powder. The elemental composition of the jujube branch powder was analyzed using an organic elemental analyzer (UNICUBE, Dresden, Germany), with the results as follows: carbon

(C) 47.21%, hydrogen (H) 5.90%, oxygen (O) 41.35%, and nitrogen (N) 0.42%.

Table S3. Jujube branch chemical composition.

Chemical Component	C	H	O	N	Other Metals
Content (%)	47.21	5.90	41.35	0.42	5.10

Table S4. Adsorption capacities: Zn/Fe-BC vs. other biochars.

Adsorbent (Biochar)	Q_e (mg/g)	References
Magnetic nanomodified sugarcane bagasse	2.5	[1]
Oxidized biochar (OBC4)	17.47	[2]
Ball-milled Fe0 –biochar composite	14.59	[3]
CTAB modified magnetic nanoparticles	18.5	[4]
Walnut shell	17.72	[5]
Cotton Stalk	15.56	[5]
Rice husk magnetic biochar	9.97	[6]
Lamb bone magnetic biochar	30.12	[7]
Zn/Fe-BC	37.658	This study

During the experiment, no color change was observed in the residual aqueous solution after the reaction (it remained clear all the time), indicating that there was no

obvious leaching of particles or colored components visible to the naked eye. To quantify the leaching extent of organic components, we measured the total organic carbon (TOC) content of the solution before and after the reaction (Table S5). In blank distilled water and unreacted Cr(VI) solution, the TOC concentration was only 0.020~0.022 mg/L. In the solution after reaction with Zn/Fe-BC, the TOC slightly increased to 0.033 mg/L, with a very small increase (only 0.011 mg/L). This result indicates that the carbon - containing components in the material (such as the biochar skeleton and surface organic functional groups) did not undergo significant leaching, and the organic stability was good.

Table S5. Determination results of total carbon (TC), inorganic carbon (IC), and total organic carbon (TOC) in different solutions.

Sample	TC (mg/L)	IC (mg/L)	TOC (mg/L)
Distilled water-1	0.083	0.061	
Distilled water-2	0.170	0.148	0.022
Distilled water-3	0.173	0.150	
50mg/L Cr(VI)-1	0.826	0.804	
50mg/L Cr(VI)-2	0.685	0.666	0.020
50mg/L Cr(VI)-3	0.776	0.755	
Post reaction Cr(VI) solution-1	2.635	2.616	
Post reaction Cr(VI) solution-2	2.624	2.561	0.033
Post reaction Cr(VI) solution-3	2.684	2.667	

Table S6. Comparison of preparation costs of different Cr(VI) removal adsorbents

Core Adsorbent	Adsorbent Preparation Cost (USD/kg)	References
zinc-doped nickel ferrite nano-adsorbent	\$36.08/1kg (literature-derived value)	[8]
CTAB–intercalated molybdenum disulfide nanosheets	\$12.79/1kg (literature-derived value)	[9]
activated biochar nanoparticles	\$15.55/1kg (literature-derived value)	[10]
Nanomagnetic Carbon Materials	\$19.48/1kg (literature-derived value)	[11]
Zn/Fe-BC	\$6.97/1kg (derived value)	This study

As can be seen from Figure S1, the blue curve representing the Cr(VI) stock solution shows obvious and relatively strong absorption peaks in the wavelength range of 200~500 nm, which indicates that the content of Cr(VI) in the stock solution is relatively high, and it has a strong characteristic absorption of ultraviolet–visible light. In contrast, the curve representing the Cr(VI) solution after the reaction has a significantly lower overall absorption intensity than that of the stock solution, and the absorbance decreases sharply at all wavelengths. This shows that after the reaction, the content of Cr(VI) in the solution is significantly reduced, indicating that Zn/Fe-BC has a good removal effect on Cr(VI).

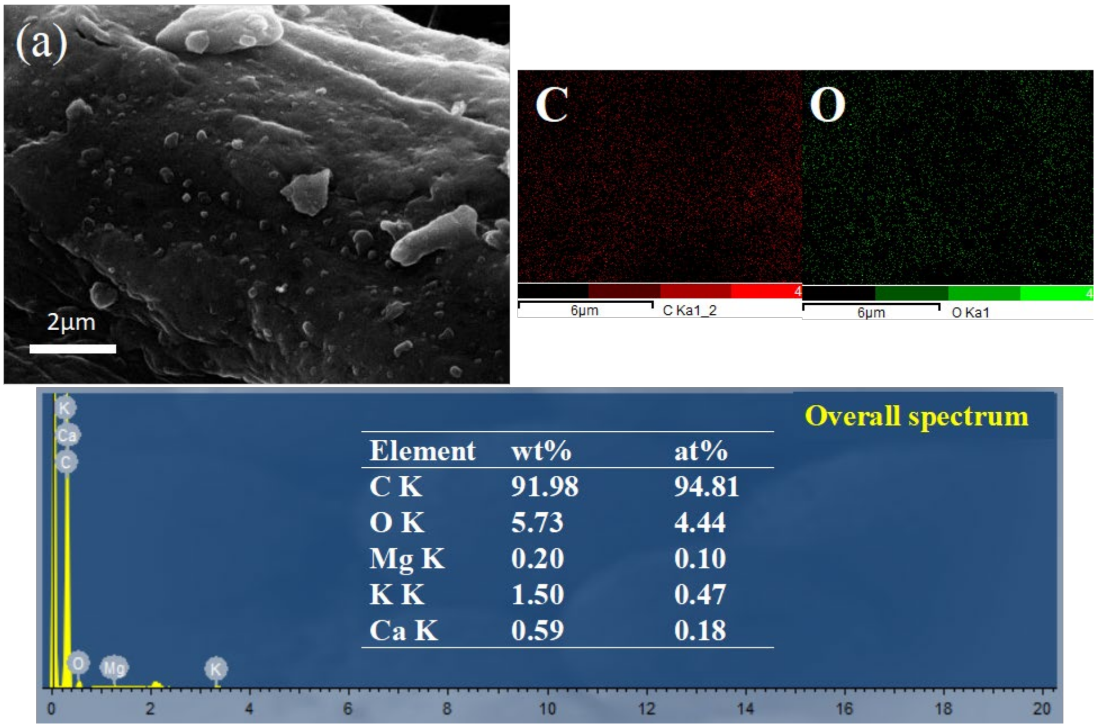

Element	wt%	at%
C K	91.98	94.81
O K	5.73	4.44
Mg K	0.20	0.10
K K	1.50	0.47
Ca K	0.59	0.18

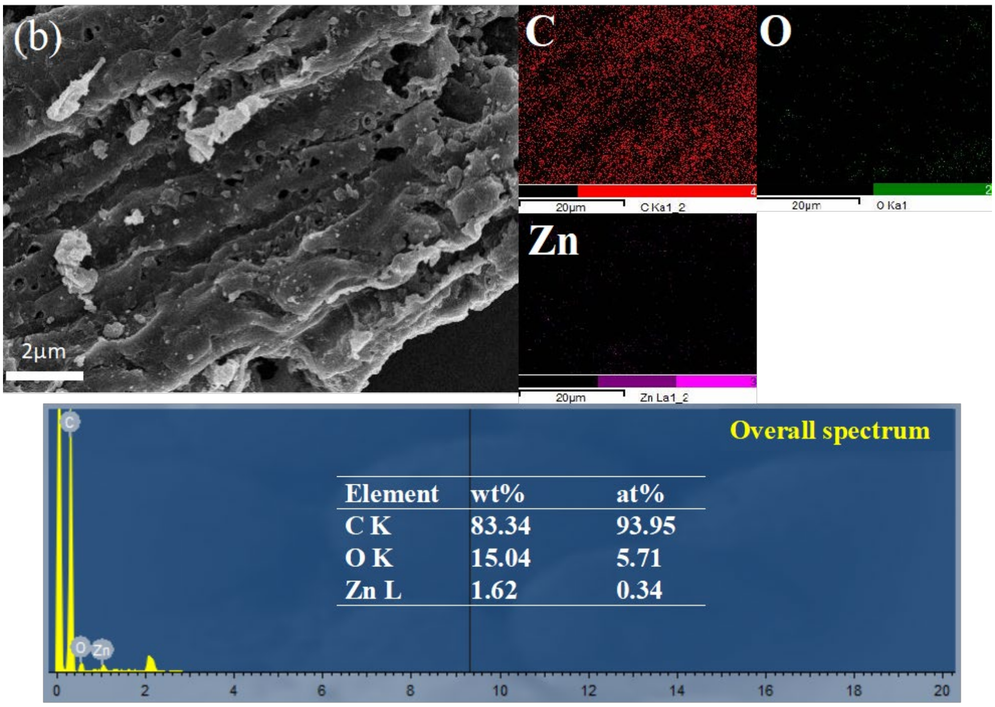

Element	wt%	at%
C K	83.34	93.95
O K	15.04	5.71
Zn L	1.62	0.34

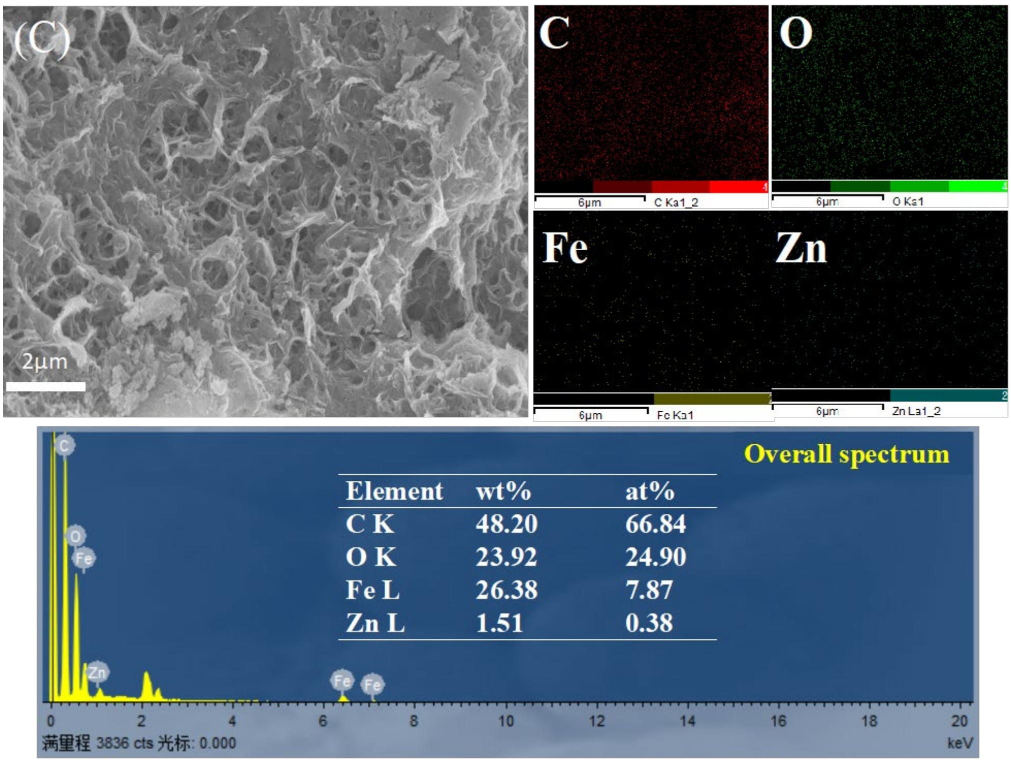

Figure S1. SEM image, element distribution, and EDS spectrum of (a)BC, (b)ZnBC, and (c)Zn/Fe-BC

To further assess the leaching risk of heavy metal components (Fe and Zn), we utilized ICP-OES to quantify their concentrations in the aqueous solution after the reaction (Table S4). The results showed that the Fe concentration in the solution was only 0.8019 mg/L, and the Zn concentration was 2.0449 mg/L. Among them, the Fe concentration is far below the limit of 10 mg/L specified in the Integrated Wastewater Discharge Standard (GB 8978 - 1996); although the Zn concentration is close to the limit of 2 mg/L in the standard, it is still within a controllable range.

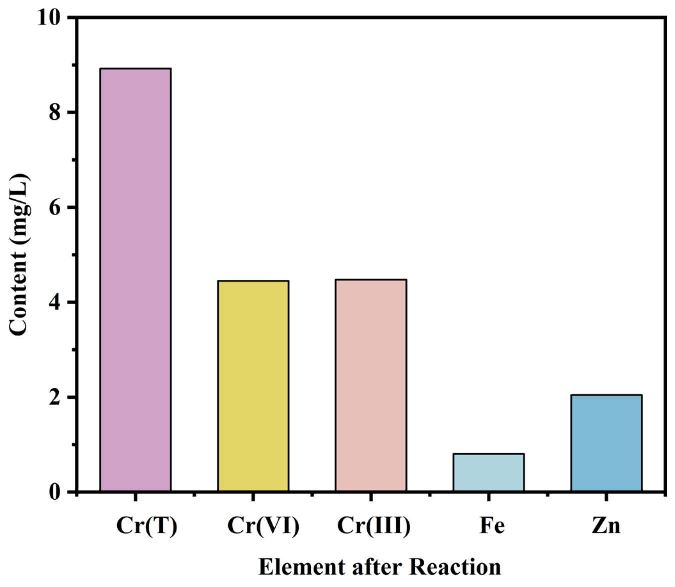

Figure S2. Distribution of element contents after reaction

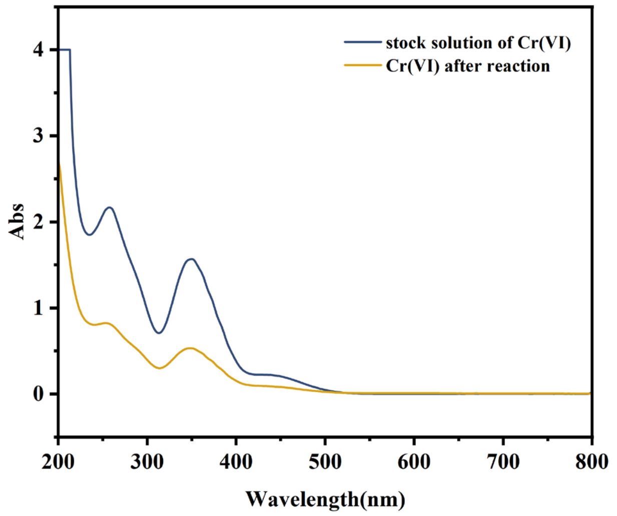

Figure S3. UV - Vis absorption spectra of Cr(VI) stock solution and solution after reaction.

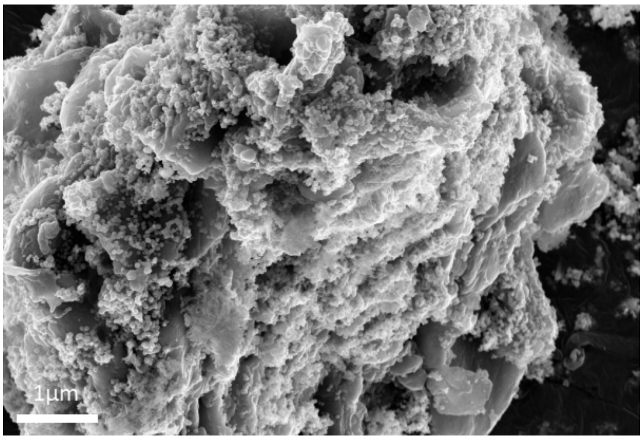

Figure S4. SEM images of Zn/Fe-BC

References

[1]T.E. Abilio, B.C. Soares, J.C. Jos´e, P.A. Milani, G. Labuto, E.N.V.M. Carrilho, Hexavalent chromium removal from water: adsorption properties of in natura and magnetic nanomodified sugarcane bagasse, Environ. Sci. Pollut. Res. 28 (19) 24816–24829, https://doi.org/10.1007/s11356-020-11726-8.

[2]Dahiya A, Bhardwaj A, Rani A, et al. Reduced and oxidized rice straw biochar for hexavalent chromium adsorption: Revisiting the mechanism of adsorption[J]. Heliyon, 2023, 9(11), https://doi.org/10.1016/j.heliyon.2023.e21735.

[3]K. Wang, Y. Sun, J. Tang, J. He, H. Sun, Aqueous Cr(VI) removal by a novel ball milled Fe0-biochar composite: role of biochar electron transfer capacity under high pyrolysis temperature, Chemosphere 241 (2020), 125044, https://doi.org/10.1016/j.chemosphere.2019.125044.

[4]S.A. Elfeky, S.E. Mahmoud, A.F. Youssef, Applications of CTAB modified magnetic nanoparticles for removal of chromium (VI) from contaminated water, J. Adv. Res. 8 (4) 435 – 443, https://doi.org/10.1016/j.jare.2017.06.002.

[5]J. Wan, F. Liu, G. Wang, W. Liang, C. Peng, W. Zhang, K. Lin, J. Yang, Exploring different mechanisms of biochars in removing hexavalent chromium: sorption, reduction and electron shuttle, Bioresour. Technol. 337 (2021), 125382, https://doi.org/10.1016/j.biortech.2021.125382.

[6]R. Sinha, et al., Single-step synthesis of activated magnetic biochar derived from rice husk for hexavalent chromium adsorption: equilibrium mechanism, kinetics, and thermodynamics analysis, Groundw. Sustain. Dev. 18 100796, https://doi.org/10.1016/j.gsd.2022.100796.

[7]D. Prabu, et al., Feasibility of magnetic nano adsorbent impregnated with activated carbon from animal bone waste: application for the chromium (VI) removal, Environ. Res. 203 111813, https://doi.org/10.1016/j. envres.2021.111813.

[8]Masuku M, Nure J F, Atagana H I, et al. The development of zinc-doped nickel ferrite nano-adsorbent for the adsorption of chromium (VI) from wastewater[J]. Journal of Water Process Engineering, 2024, 64: 105587.

[9]Cai W, Dionysiou D D, Fu F, et al. CTAB–intercalated molybdenum disulfide nanosheets for enhanced simultaneous removal of Cr (VI) and Ni (II) from aqueous solutions[J]. Journal of Hazardous Materials, 2020, 396: 122728.

[10]Dai X, Luo Y, Deng J, et al. Ultra-efficient removal of aqueous hexavalent chromium by activated biochar nanoparticles derived from squid ink[J]. Environmental Research, 2024, 263: 120185.

[11]Long W, Chen Z, Chen X, et al. Investigation of the adsorption process of chromium (VI) ions from petrochemical wastewater using nanomagnetic carbon materials[J]. Nanomaterials, 2022, 12(21): 3815.
